# A case of carbapenamase production in *Shigella flexneri* isolated in Ireland, 2025

**DOI:** 10.2807/1560-7917.ES.2026.31.18.2600351

**Published:** 2026-05-07

**Authors:** Christina Clarke, Alma Tuohy, Erica Duignan, Paul Mullane, Ann Marie Carolan, Orla Craig, Georgios Miliotis, Christina Dillon, Martin Cormican

**Affiliations:** 1 Galway Reference Laboratory Service, Galway University Hospital, Galway, Ireland; 2 Department of Microbiology, Mater Misericordiae University Hospital, Dublin, Ireland; 3 Department of Public Health, Dublin and North East Region, Dublin, Ireland; 4 Department of Gastroenterology, Mater Misericordiae University Hospital, Dublin, Ireland; 5 School of Medicine, University of Galway, Galway, Ireland; 6 Health Protection Surveillance Centre, Dublin, Ireland

**Keywords:** *Shigella flexneri*, carbapenemase, antimicrobial resistance, blaOXA-181

## Abstract

*Shigella flexneri *producing OXA-181 was detected from a patient in Ireland in October 2025. The isolate had a meropenem minimum inhibitory concentration (MIC) of 0.12 mg/L. Carbapenemase production was detected by lateral flow immunoassay. The sequence type was ST245 and closely related to sequences previously reported from Ireland and other countries but not previously linked to presence of the *bla*_OXA-181_ gene_._ Carbapenemase production in *Shigella* spp. has implications for laboratory detection and empiric treatment.

Antimicrobial-resistant *Shigella* spp. infection is a growing public health problem, and although shigellosis is often self-limiting, some people develop serious infection requiring antimicrobial treatment. Resistance to third-generation cephalosporins, ciprofloxacin and azithromycin may require the use of carbapenems as initial empiric therapy in patients with serious infection [1,2]. In October 2025, we identified a case of infection with a carbapenemase (OXA-181)-producing *Shigella flexneri* sequence type (ST) 245 in a resident of Ireland with recent travel to Brazil and Spain. Here, we report the clinical and genomic analyses of the case. To our knowledge, this is the first case of carbapenemase (OXA-181)-producing *Shigella* detected in the European Union/European Economic Area (EU/EEA).

## Clinical case description

The patient, a male in his 50s, presented in October 2025 with acute onset of vomiting and diarrhoea. He is resident in Ireland but had travelled to Brazil (departing from Brazil 3 days before symptom onset) and Spain (departing from Spain on the day of symptom onset). He had not eaten street food while travelling, but several hours prior to symptom onset, he reported eating chicken in the airport in Barcelona that tasted bad. He also reported that a friend in Spain had been unwell with salmonellosis. The patient identified as a man who has sex with men (MSM), but reported no sexual contact while travelling in Brazil and Spain.

Six days after onset of symptoms, he was admitted to the hospital. At peak of symptom severity, about the time of hospital admission, the patient had a bowel movement every 20 minutes with episodes of passing small amounts of blood. He had developed fever (39°C) and rigors at home, experiencing weight loss of 7 kg in the previous week. At hospital admission, he was dehydrated and continued to have diarrhoea. Blood urea was 10 mmol/L (reference range: 2.8–8.6) and creatinine was 129 µmol/L (reference range: 65–107); C-reactive protein (CRP), white blood cell count, ALP (alkaline phosphatase), and GGT (gamma-glutamyl transferase) were all above reference ranges. Computed tomography (CT) imaging showed pancolitis. He received intravenous fluid, analgesics and anti-emetics. Diarrhoea persisted on the day after hospital admission.

Empiric antimicrobial treatment for possible *Shigella* infection was then commenced with oral ciprofloxacin (500 mg every 12 hours) and azithromycin (500 mg daily). In the context of frequent occurrence of antimicrobial resistance in *Shigella*, the two agents were administered empirically to increase the likelihood that the patient was receiving an agent active against the pathogen causing infection. Treatment duration was 3 days. There was gradual symptom resolution over the subsequent 3–4 days. The patient was hospitalised for a total of 7 days. At discharge on day 7 after hospital admission, CRP, white cell count, GGT, urea and creatinine were in the reference range. ALP remained above the reference range.

## Microbiological findings 

*Shigella* spp. were isolated from a faecal sample collected on the day of hospital admission. The isolate was submitted to the National Reference Laboratory Service at Galway University Hospital and subjected to short read whole genome sequencing (Illumina). The isolate was confirmed as *S. flexneri* ST245 (Bruker’s MBioSeqRidom Typer), and found to carry the *bla*_OXA-181_ gene.

**Figure 1:**
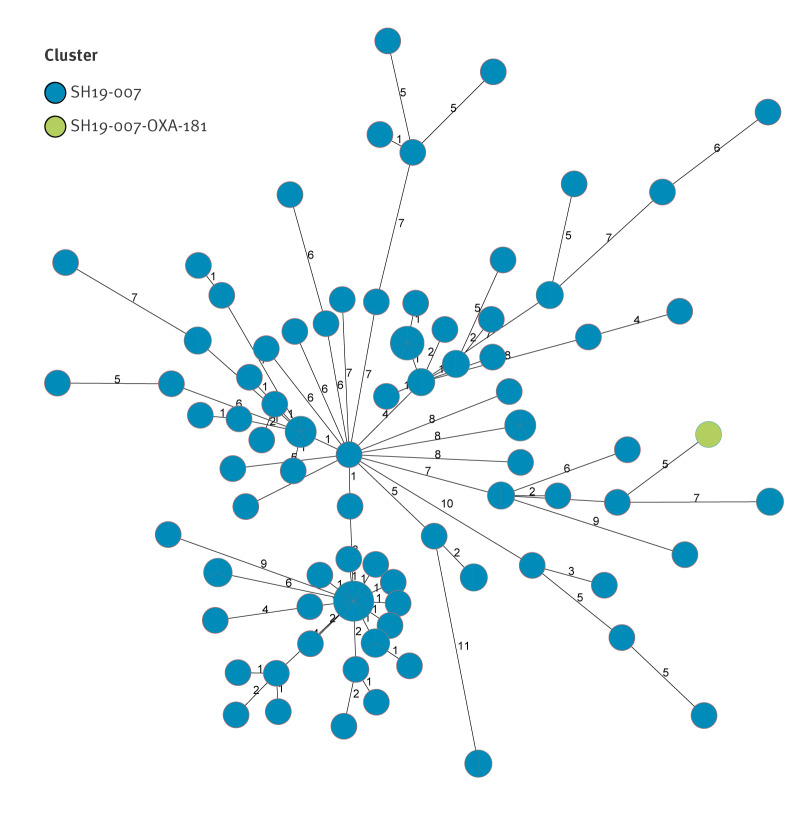
Minimum spanning tree of a cluster of *Shigella flexneri* isolated in Ireland, June 2017–April 2026 (n = 117)

**Figure 2:**
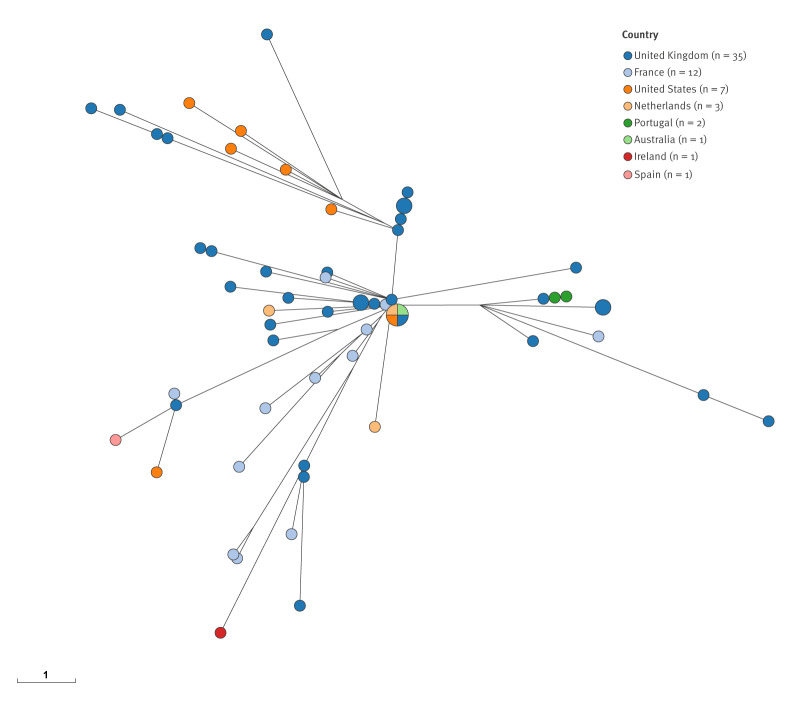
Minimum spanning tree of closely related *Shigella flexneri* isolates, Europe and worldwide, 29 June 2017–6 May 2026 (n = 62)

On core genome MLST, the isolate clustered with a group of 117 other isolates detected in Ireland since 2019 (up to 15 April 2026; strain designated SH19-007) (Figure 1). This diffuse cluster represents 27% of 437 non-duplicate *S. flexneri *isolates received during this period. This strain has previously been associated with MSM. On the EnteroBase database, there are strains from seven other countries (Australia, France, Netherlands, Portugal, Spain, United Kingdom and United States) that cluster with this strain at a HC05 hierarchical threshold (less than 5 allelic differences from other isolates in the tree) (Figure 2). 

The minimum spanning tree shows a cluster of *Shigella flexneri*, designated SH19-007, isolated in Ireland between 29 June 2017 and 15 April 2026 including the isolate described here based on core genome MLST coloured differently to other isolates. The number adjacent to lines indicate number of allelic differences between nodes. Divided nodes represent multiple isolates indistinguishable at cgMLST level. The figure was generated with MBioSEQ Ridom Typer.

The minimum spanning tree shows *Shigella flexneri *isolates within 5 allelic differences (AD) of each other. The *S. flexneri* isolate reported here is indicated by the red node at bottom of figure. Nodes are coloured by the country associated with the sequence; the number of sequences per country is indicated in parentheses. The distance between nodes represents number of allelic differences between isolate sequences. The figure was generated using sequences publicly available on EnteroBase on 6 May 2026 using GrapeTree version 1.5.0.

Enzyme production was detectable with the NG-BIOTECH Laboratories NG-Test/CARB-5 lateral flow device. In addition to *bla*_OXA-181_, the strain carried antimicrobial resistance genes including *cat*A1, *qnrS1*, *tet*(B), *dfr*A1 and an *Inc*X3 replicon. Minimum inhibitory concentrations (MIC) were meropenem 0.12 mg/L (susceptible), ampicillin > 32 mg/L (resistant), chloramphenicol 64 mg/L (consistent with acquired resistance), cefotaxime 0.5 mg/L (susceptible), azithromycin 4 mg/L (consistent with wild type) and ciprofloxacin 0.5 mg/L (area of technical uncertainty, ATU). Prior to detection of this isolate, the reference laboratory had characterised 519 other Enterobacterales with *bla*_OXA-181_ and an *Inc*X3 replicon over the past 9 years; however, this is the first detection of *bla*_OXA-181_ in *Shigella *spp.

## Discussion

Antimicrobial-resistant shigellosis is a public health problem in Europe. Most shigellosis in the EU/EEA is caused by *S. flexneri* or *S. sonnei*. Transmission of *Shigella* spp. is faecal-oral through contaminated hands, food and fomites. It is also an important sexually transmitted enteric infection (STEI) particularly impacting MSM. A European Centre for Disease Prevention and Control (ECDC) Rapid Risk Assessment on extensively drug-resistant *S. sonnei* in MSM in 2022 and a subsequent update in 2023 highlight the growing concerns regarding antimicrobial resistance [1,3]. A similar phenomenon is reported in the United States [4]. Although shigellosis is often a self-limiting gastrointestinal disease, some patients develop serious infection including bacteraemia. Acquired antimicrobial resistance in *S. flexneri* and *S. sonnei* have led some authorities to recommend a carbapenem for the initial empiric treatment of seriously ill patients with suspected or confirmed shigellosis [2].

Carbapenemase-producing *Shigella* spp. have been reported previously. Detection of the *bla*_OXA-181_ gene in *S. flexneri* was reported from Canada in 2024 from a 2-year-old immunocompromised child born in India [5]. The isolate was categorised as susceptible to meropenem with a MIC of 0.5 mg/L. There are previous reports of metallo-carbapenemase- producing *Shigella* from Egypt (2022, [6]) and the Andaman Islands (2015, [7]).

The European Committee for Antimicrobial Susceptibility Testing (EUCAST) criteria for interpretation of meropenem susceptibility test results for Enterobacterales are susceptible at ≤ 2 mg/L or zone diameter ≥ 22 mm [8]. Enterobacterales-producing carbapenemases, in particular *bla*_OXA-181_ and other *bla*_OXA48_-like carbapenemases, are frequently categorised susceptible to carbapenems. When screening for carbapenemase production, EUCAST recommends a screening cut-off for meropenem of > 0.12 mg/L and a zone diameter of < 28 mm. Detection of carbapenemase production in carbapenem-susceptible Enterobacterales is important. EUCAST guidance indicates that if a carbapenemase is detected, the clinical response to treatment with carbapenems may be impaired even if the isolate is categorised as susceptible (S) or susceptible, increased exposure (SIE).

In this case, the isolate was wild type for azithromycin, the MIC for ciprofloxacin was in the ATU [8] and the patient responded well to antimicrobial treatment. However, *S. flexneri* resistant to azithromycin and fluoroquinolones is now also commonly detected; therefore, reliable options for initial empiric treatment are increasingly limited [1,3]. The emergence of carbapenemase *bla*_OXA-181_ in *S. flexneri* poses challenges for treatment and for detection. OXA-181-producing isolates often test susceptible to meropenem as did this isolate, and in this case, the MIC was below the EUCAST screening cut-off for carbapenemase. Testing for antigen production by lateral flow immunoassay may provide a rapid and convenient approach to test for carbapenemase production. The kit insert for the NG CARBA-5 indicates that it detects *bla*_OXA-181_ as an OXA-48-like carbapenemase. Experience in our laboratory is consistent with this. Consultation with clinical microbiology or infectious disease specialists may assist in managing suspected or confirmed cases of shigellosis with serious illness given the increasing challenges with acquired resistance.

The origin of this strain is unclear. The timing of travel and onset of symptoms suggests travel-related infection. However, the *S. flexneri* strain (SH19-007) is established in Ireland, as in other countries, and *bla*_OXA-181_ is established in other Enterobacterales in Ireland. It is possible that the described carbapenemase-producing *S. flexneri* emerged by transfer of a mobile genetic element from another species of Enterobacterales.

## Conclusion

It is important for laboratories and treating clinicians to be aware of carbapenemase production in *Shigella *spp. and that such strains may be missed by phenotypic susceptibility testing. 

## Data Availability

Sequence data generated in this study have been shared via the European Nucleotide Archive and can be retrieved as ENA accession number: ERR15905248.

## References

[1] European Centre for Disease Prevention and Control (ECDC). Rapid risk assessment: Increase in extensively-drug resistant Shigella sonnei infections in men who have sex with men in the EU/EEA and the UK – 23 February 2022. Stockholm: ECDC; 2022. Available from: https://www.ecdc.europa.eu/en/publications-data/rapid-risk-assessment-increase-extensively-drug-resistant-shigella-sonnei

[2] Health Service Executive (HSE) Public Health-Health Protection. Recommendations on aspects of management of shigellosis in Ireland in the context of current antimicrobial resistant Shigella species associated with gay, bisexual and men who have sex with men (gbMSM) Version 1. Dublin: HSE Public Health-Health Protection; 2024. Available from: https://www.hpsc.ie/a-z/gastroenteric/shigellosis/guidancepublications/Recommendations%20of%20aspects%20of%20management%20of%20shigellosis.pdf

[3] European Centre for Disease Prevention and Control (ECDC). Spread of multidrug-resistant Shigella in EU/EEA among gay, bisexual and other men who have sex with men. – 18 July 2023. Stockholm: ECDC; 2023. Available from: https://www.ecdc.europa.eu/en/news-events/spread-multidrug-resistant-shigella-eueea-among-gay-bisexual-and-other-men-who-have-sex

[4] Logan N Birhane MG McDonald SL Alarcón J Amador S Anand M XDR Shigella Working Group. Emergence of extensively drug-resistant shigellosis — United States, 2011–2023. MMWR Morb Mortal Wkly Rep. 2026;75(13):173-8. 10.15585/mmwr.mm7513a1 41955161 PMC13065089

[5] Dhabaan G Jamal H Ouellette D Alexander S Arane K Campigotto A Detection of OXA-181 carbapemenase in Shigella flexneri. Emerg Infect Dis. 2024;30(5):1048-50. 10.3201/eid3005.231558 38666725 PMC11060442

[6] Abdelaziz NA. Phenotype-genotype correlations among carbapenem-resistant Enterobacterales recovered from four Egyptian hospitals with the report of SPM carbapenemase. Antimicrob Resist Infect Control. 2022;11(1):13. 10.1186/s13756-022-01061-7 35063019 PMC8783469

[7] Thamizhmani R Rhagavan R Sugunan AP Vijayachari P. VIM- and IMP-type metallo-β-lactamase-producing Shigella spp. in childhood diarrhea from Andaman Islands. Infect Dis (Lond). 2015;47(10):749-50. 10.3109/23744235.2015.1022874 25768231

[8] European Committee on Antimicrobial Susceptibility Testing (EUCAST). Clinical Breakpoint Tables. Version 16.0. Växjö: EUCAST; 2025. Available from: https://www.eucast.org/bacteria/clinical-breakpoints-and-interpretation/clinical-breakpoint-tables

